# Supplementary material for: Tin(II) and Tin(IV) Complexes Incorporating the Oxygen Tripodal Ligands [(η5-C5R5)Co{P(OEt)2O}3]−, (R = H, Me; Et = -C2H5) as Potent Inflammatory Mediator Inhibitors: Cytotoxic Properties and Biological Activities against the Platelet-Activating Factor (PAF) and Thrombin
Source: Molecules. 2023 Feb 16;28(4):1859. doi: 10.3390/molecules28041859 (PMC9964123; doi:10.3390/molecules28041859)
Supplement: Supplementary file 1 [file molecules-28-01859-s001.zip › molecules-2201802-supplementary.pdf]

Supplementary material

For

Tin(II) and tin(IV) complexes incorporating the oxygen tripodal ligand [(η^5-C_5R_5)Co{P(OEt)$_2$O}$_3$]$^-$, (R = H, Me; Et = -C_2H_5): Cytotoxic properties and biological activities against the Platelet-Activating Factor (PAF) and Thrombin

Alexandros Kalampalidis [1], Artemis Damati [2,3], Demetrios Matthopoulos [2], Alexandros B. Tsoupras [4,5,6], Constantinos Demopoulos [7], Gregor Schnakenburg [8], and Athanassios Philippopoulos [1,*]

[1] Laboratory of Inorganic Chemistry, Department of Chemistry, National and Kapodistrian University of Athens, Panepistimiopolis Zografou 15771, Athens, Greece

[2] Department of Environmental Engineering. School of Engineering. University of Patras, Greece

[3] Department of Forestry and Environment Natural Management. School of Plant Sciences, Agricultural University of Athens, Greece

[4] Department of Biological Sciences, University of Limerick, Limerick V94 T9PX, Ireland

[5] Health Research Institute, University of Limerick, Limerick V94 T9PX, Ireland

[6] Bernal Institute, University of Limerick, Limerick V94 T9PX, Ireland

[7] Laboratory of Biochemistry, Department of Chemistry, National and Kapodistrian University of Athens, Panepistimiopolis Zografou 15771, Athens, Greece

[8]Institut für Anorganische Chemie, Rheinische Friedrich-Wilhelms-Universität Bonn, Gerhard-Domagk-Straße 1, D-53121 Bonn, Germany

* Corresponding author: Prof. A. I. Philippopoulos. Tel.: +30-210-7274697; e-mail: atphilip@chem.uoa.gr

Table of Contents

Entry

**1) Figure S1**	FT-IR (ATR mode) spectrum of **2a**
**2) Figure S2**	FT-IR (ATR mode) spectrum of **2b**
**3) Table S1**	Selected bond lengths (pm) and bond angles (°) with estimated standard deviations for complexes for **2a, 2b** and **3b**
**4) Figure S3**	Hydrogen bonding interactions in **2a**
**5) Figure S4**	Hydrogen bonding interactions in **2b**
**6) Figure S5**	FT-IR spectra of **3a**
**7) Figure S6**	FT-IR spectra of **3b**
**8) Figure S7**	^{1}H NMR of **3a** in $CDCl_3$
**9) Figure S8**	^{31}P NMR of **3a** in $CDCl_3$
**10) Figure S9**	^{1}H NMR of **3b** in $CDCl_3$
**11) Figure S10**	^{31}P NMR data of **3b** in $CDCl_3$
**12) Figure S11**	C–H···π interactions in **3b**
**13) Figure S12**	^{1}H NMR spectrum of **2a** recorded in DMSO-d_6
**14) Figure S13**	^{1}H NMR spectrum of **2b** recorded in DMSO-d_6
**15) Figure S14**	^{1}H NMR spectrum of **3a** recorded in DMSO-d_6
**16) Figure S15**	^{31}P NMR spectrum of **3a** recorded in DMSO-d_6
**17) Table S2**	Viability percentage (%) values for organotin(II) and organotin(IV) complexes against the Jurkat T lymphoblastic tumor cell line
**18) entry**	Molecular docking Studies
**19) entry**	Details of the experimental protocol for biological experiments on washed rabbit platelets (WRPs)

1) Table S1

Selected bond lengths (pm) and bond angles (°) with estimated standard deviations for complexes **2a, 2b** and **3b.**

	**2a**	**2b**	**3b**		**2a**	**2b**	**3b**
Bond lengths				*Bond angles*			
Sn–Cl	258.47(10)[a]	262.4(2)		Cl–Sn–O(1)	86.96(6)	84.90(14)	
Sn–O(1)	213.8(2)	210.2(4)	220.6(5)	Cl–Sn–O(2)	84.90(6)	87.20(149	
Sn–O(2)	212.47(19)	212.0(4)	221.7(4)	Cl–Sn–O(3)	157.85(5)	158.45(11)	
Sn–O(3)	245.8(2)	238.6(5)	221.8(4)	O(1)–Sn–O(2)	89.93(8)	88.89(16)	79.31(16)
P(1)–O(1)	153.3(2)	153.9(4)	150.5(5)	O(1)–Sn–O(3)	78.68(8)	78.43(17)	79.08(17)
P(1)–O(4)	159.35(2)	161.6(5)	157.1(6)	O(2)–Sn–O(3)	78.49(7)	78.99(16)	78.31(16)
P(1)–O(5)	159.7(2)	159.7(4)	158.0(7)	P(1)–O(1)–Sn	132.10(12)	137.5(3)	135.5(3)
P(2)–O(2)	152.76(19)	153.6(4)	149.8(5)	P(2)–O(2)–Sn	135.56(12)	132.6(2)	136.2(3)
P(2)–O(6)	159.25(2)	159.4(5)	157.8(5)	P(3)–O(3)–Sn	128.58(12)	132.1(3)	136.3(3)
P(2)–O(7)	160.85(2)	160.6(5)	159.4(6)	Co–P(1)–O(1)	119.36(9)	119.25(18)	120.0(2)
P(3)–O(3)	150.0(2)	150.8(4)	150.7(5)	Co–P(1)–O(4)	109.55(11)	115.35(19)	109.4(3)
P(3)–O(8)	160.25(3)	160.3(5)	157.5(7)	Co–P(1)–O(5)	111.05(9)	107.89(19)	109.3(2)
P(3)–O(9)	159.3(2)	161.2(4)	157.4(7)	Co–P(2)–O(2)	119.89(9)	121.61(15)	120.32(19)
Co–P(1)	216.28(9)	218.39(18)	216.1(2)	Co–P(2)–O(6)	109.74(10)	106.34(18)	111.2(2)
Co–P(2)	216.34(10)	217.73(17)	215.68(19)	Co–P(2)–O(7)	110.72(11)	113.8(2)	108.9(2)
Co–P(3)	217.05(10)	219.13(19)	216.7(2)	Co–P(3)–O(3)	119.68(9)	119.3(2)	118.78(19)
Co–C(1)	207.15(4)	214.2(6)	209.0(8)	Co–P(3)–O(8)	110.68(10)	106.83(18)	109.6(3)
Co–C(2)	208.9(3)	215.1(6)	212.5(7)	Co–P(3)–O(9)	104.16(10)	111.7(2)	111.9(4)
Co–C(3)	209.55(3)	212.0(7)	211.1(7)	P(1)–Co–P(2)	93.08(4)	92.05(79	90.50(7)
Co–C(4)	207.7(4)	213.9(6)	211.0(7)	P(1)–Co–P(3)	91.62(4)	87.99(7)	89.44(8)
Co–C(5)	207.15(4)	212.1(6)	211.7(8)	P(2)–Co–P(3)	90.52(4)	91.04(7)	130.4(2)

[a] Two crystallographically independent molecules are found in the crystal structure; the listed bond lengths and angles are the average values for the two molecules.

General comment about the crystal structures reported in this work.

The X-ray structures of **2a, 2b and 3b** have been measured some years ago. This to some extent, explains the limited quality of the structure determinations of **2b** and **3b.**

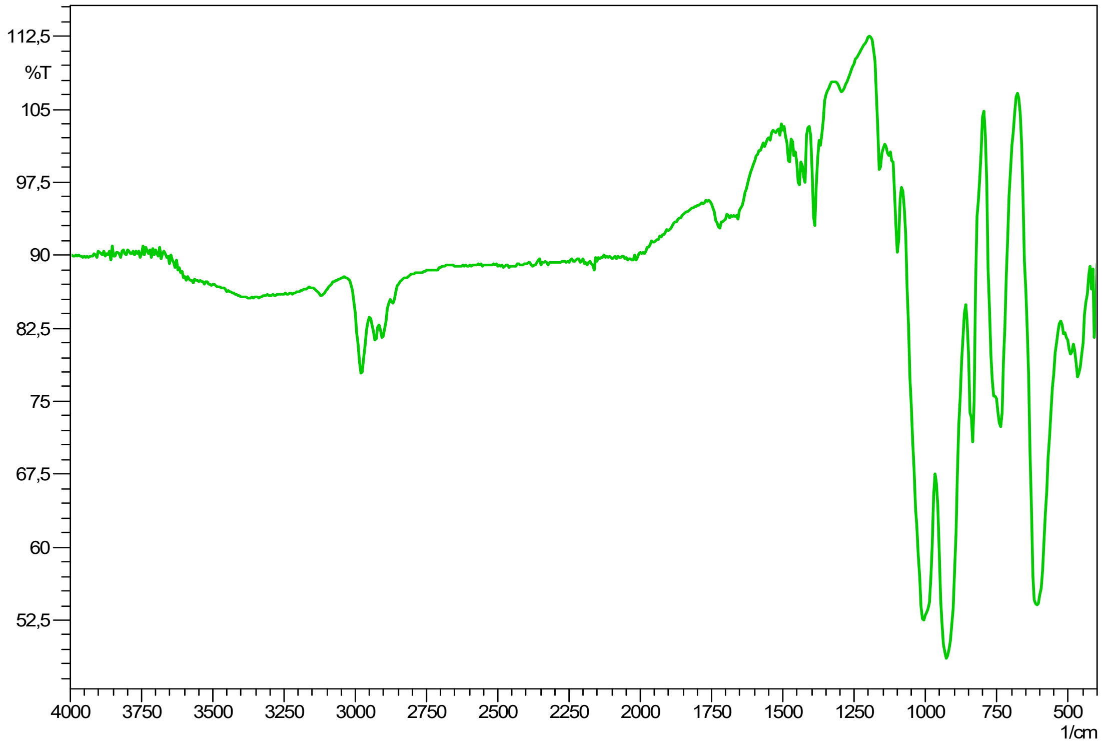

2) **Figure S1**. FT-IR (ATR mode) spectrum of **2a**

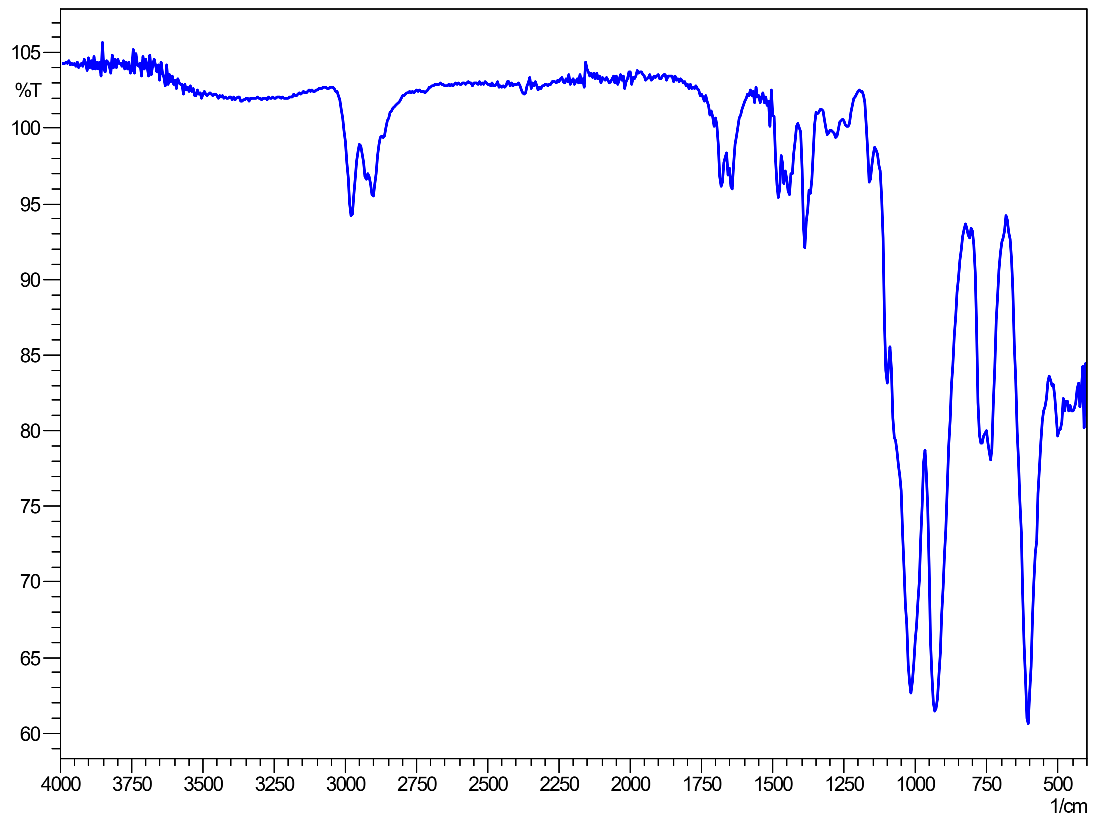

3) **Figure S2**. FT-IR (ATR mode) spectrum of **2b**

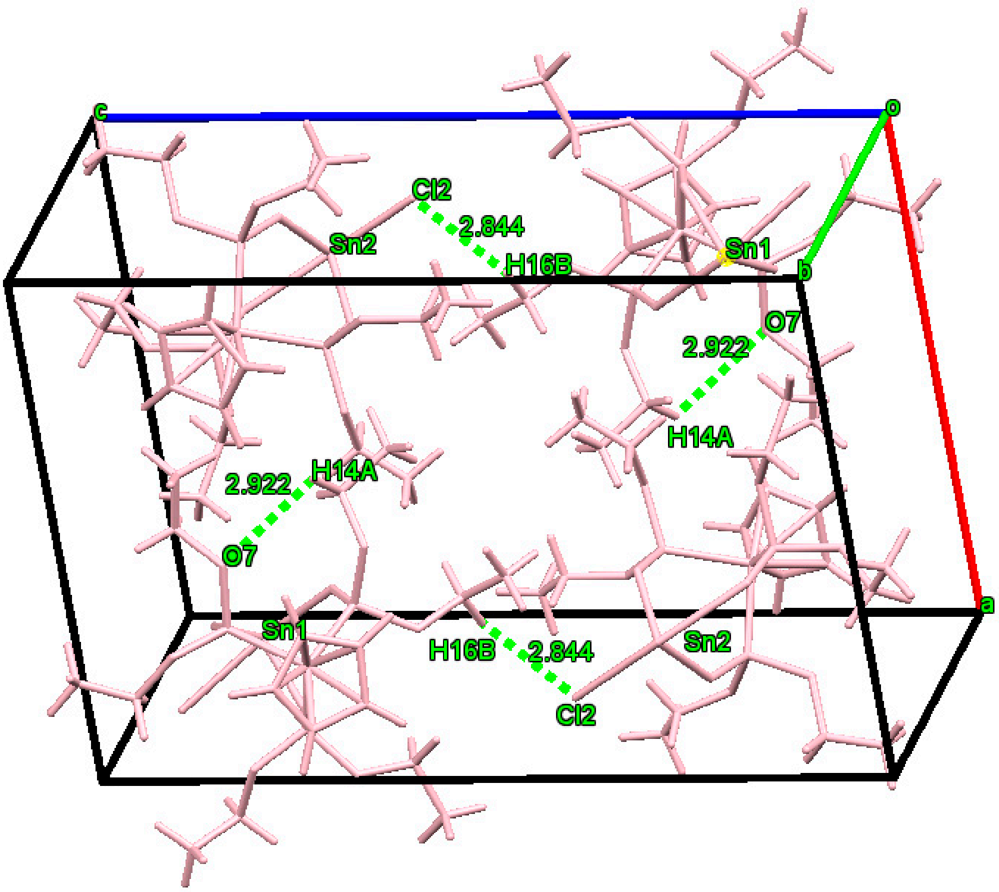

4) **Figure S3**. Hydrogen bonding interactions in **2a**

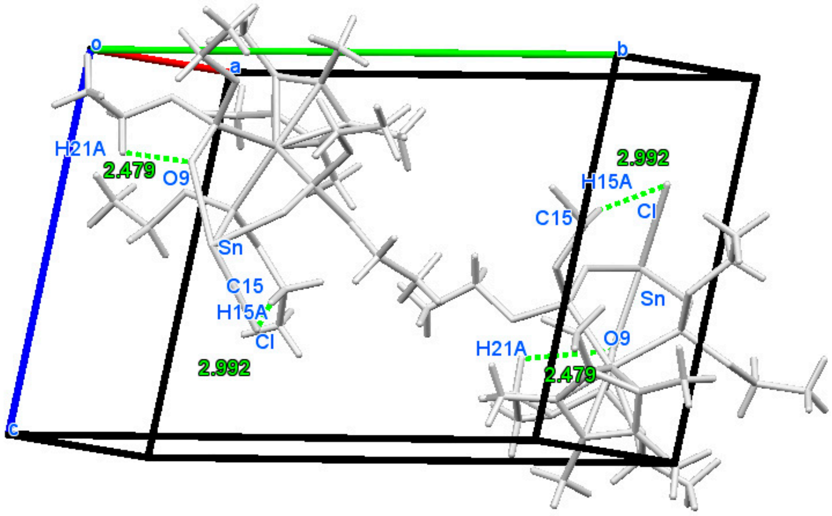

5) **Figure S4**. Hydrogen bonding interactions in **2b**

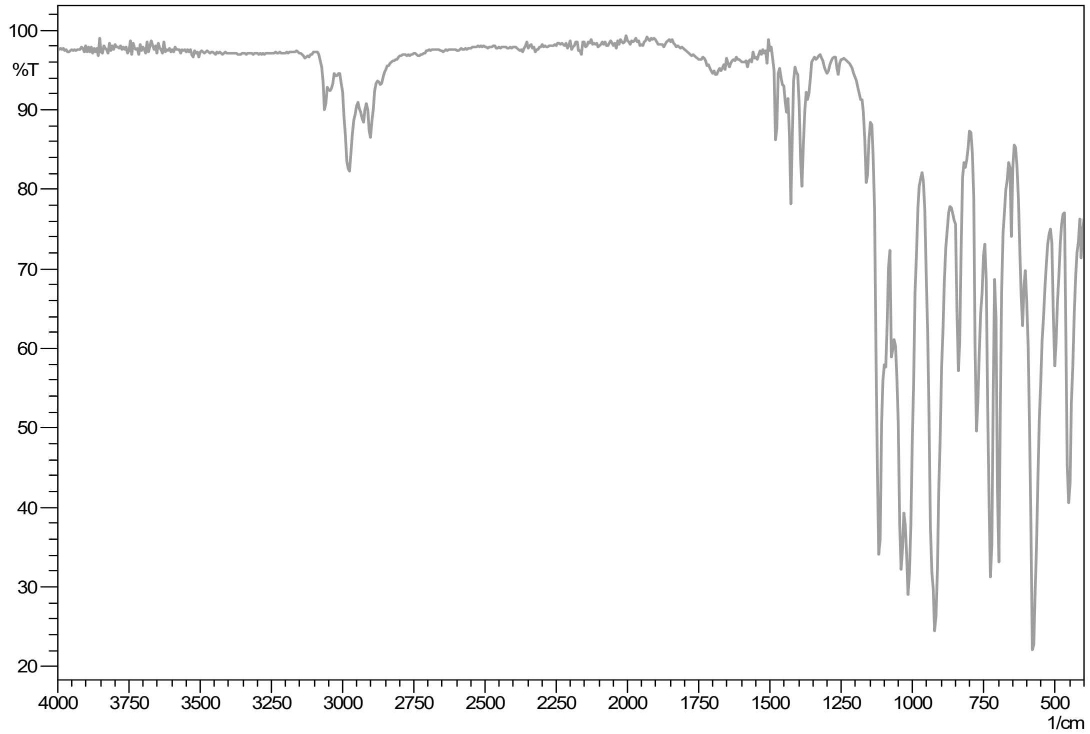

6) **Figure S5**: FT-IR spectra of **3a**

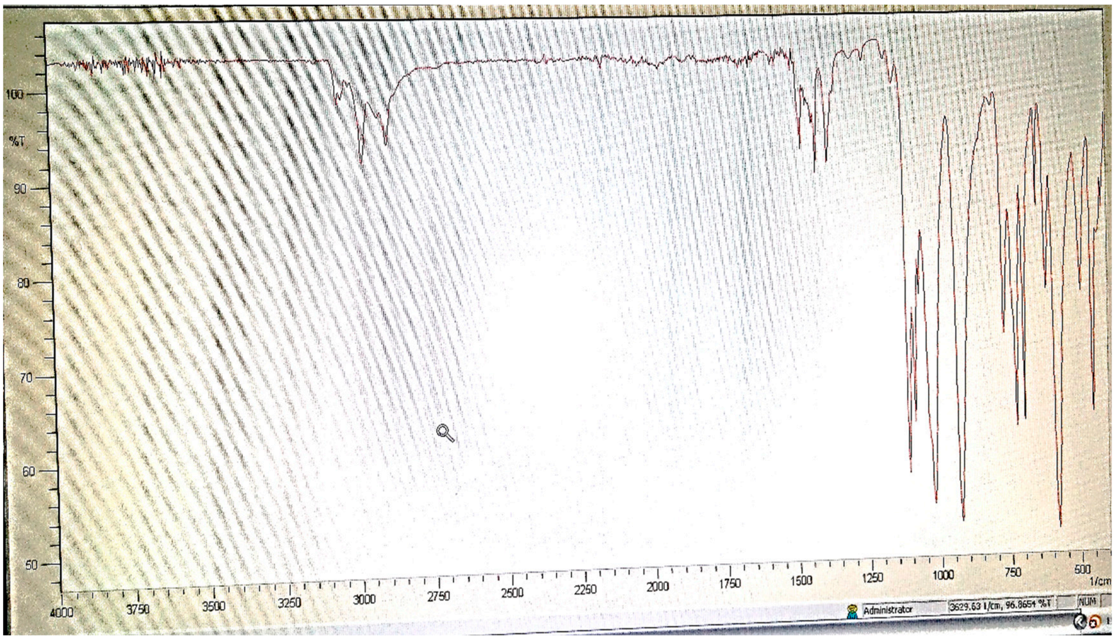

7) **Figure S6**: FT-IR spectra of **3b**

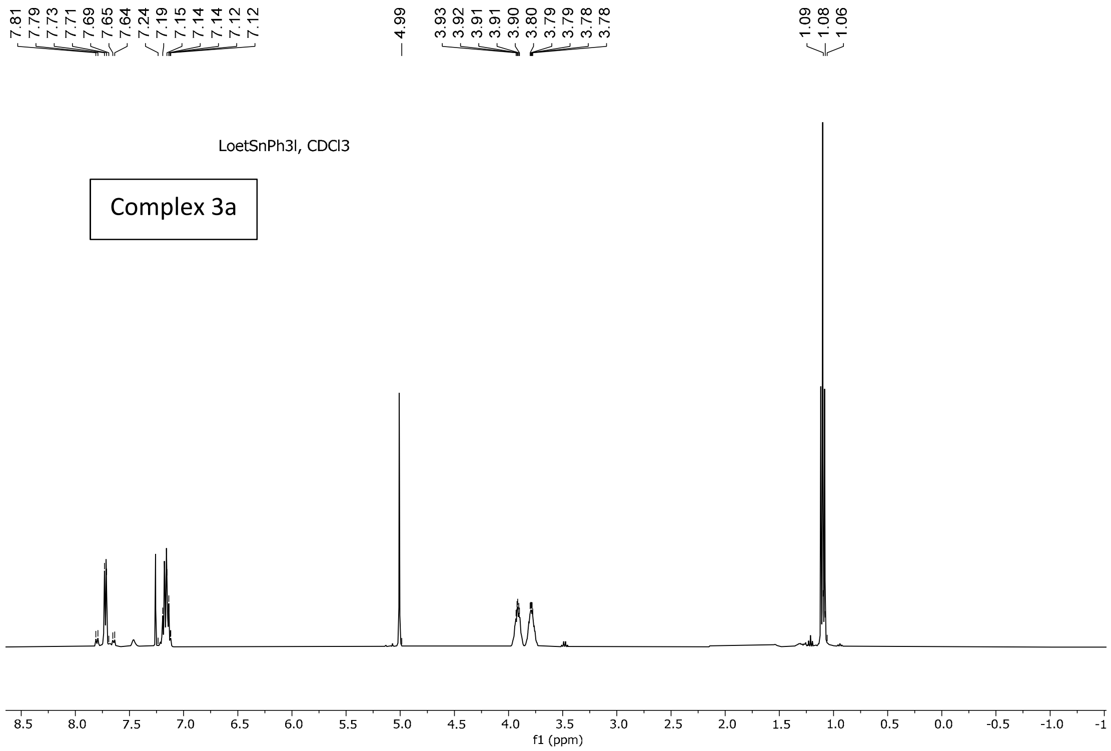

8) **Figure S7.** ^{1}H-NMR of **3a** in $CDCl_3$ (400 MHZ)

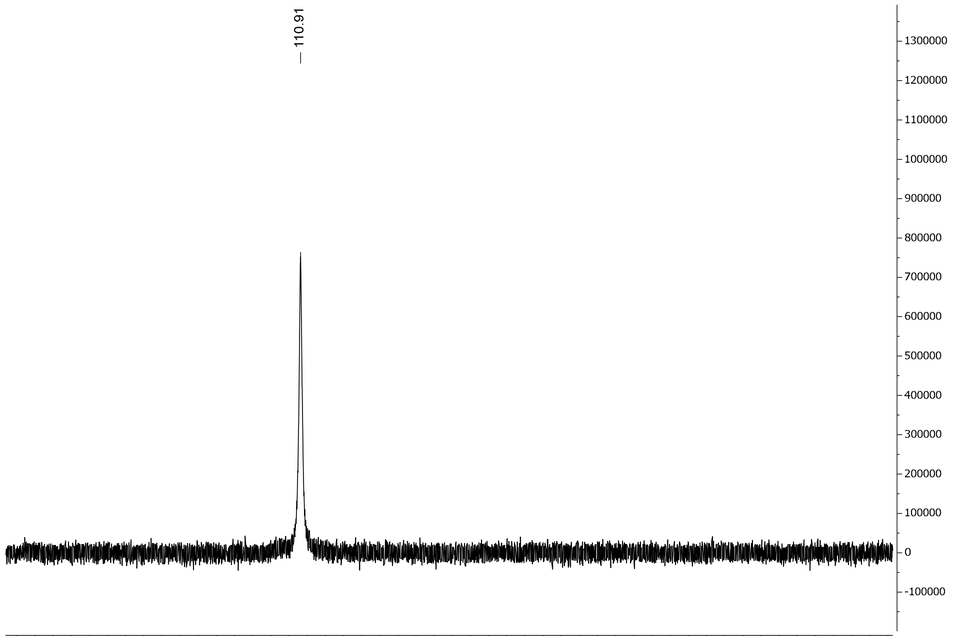

9) **Figure S8.** ^{31}P-NMR of **3a** in $CDCl_3$ (400 MHZ)

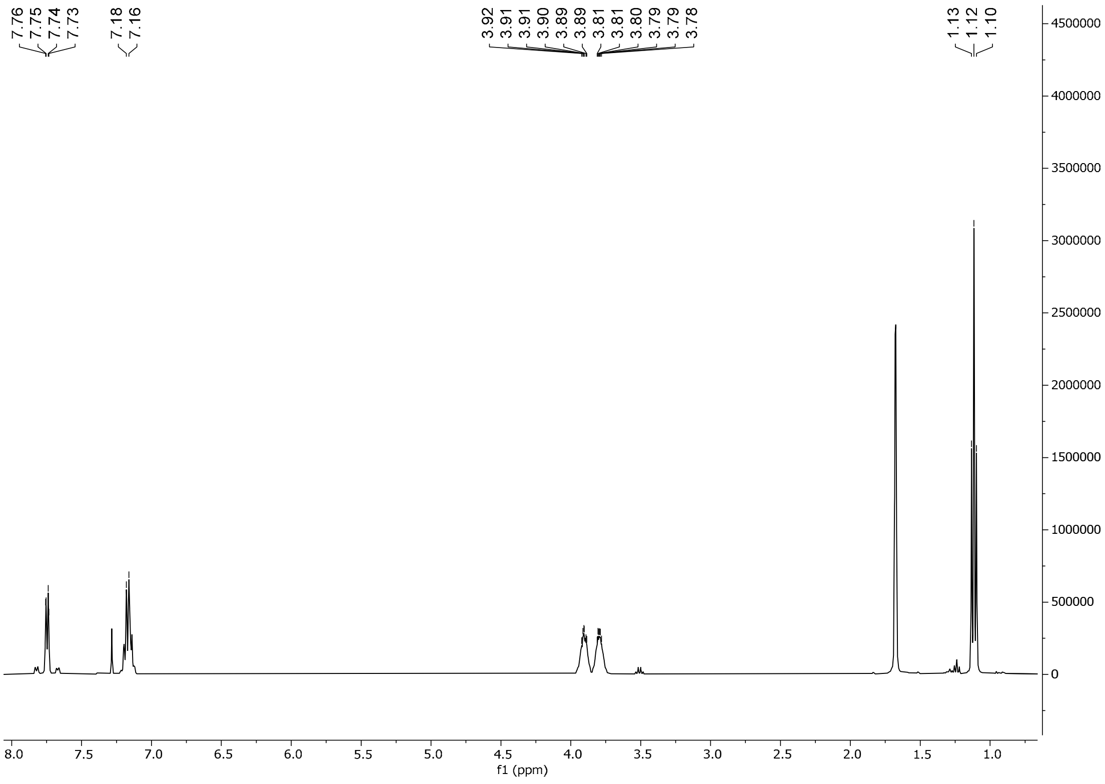

10) **Figure S9**. ^{1}H-NMR of **3b** in $CDCl_3$ (400 MHZ)

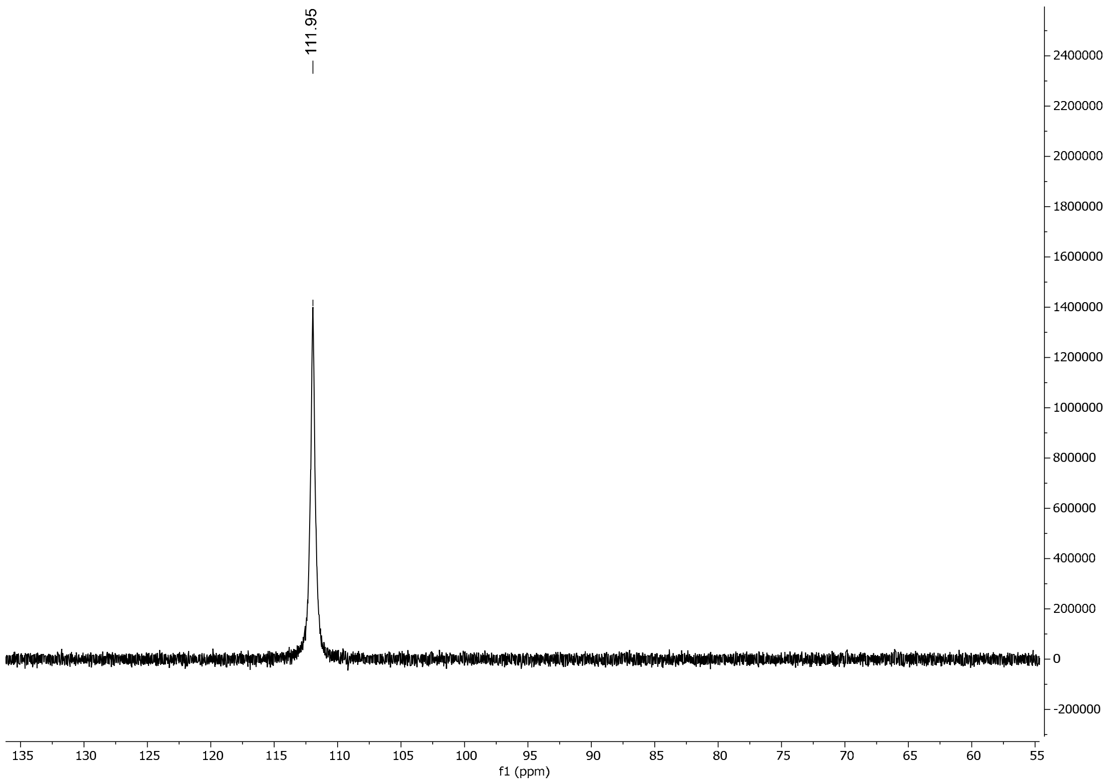

11)**Figure S**10. ^{31}P-NMR of **3b** in $CDCl_3$ (400 MHZ)

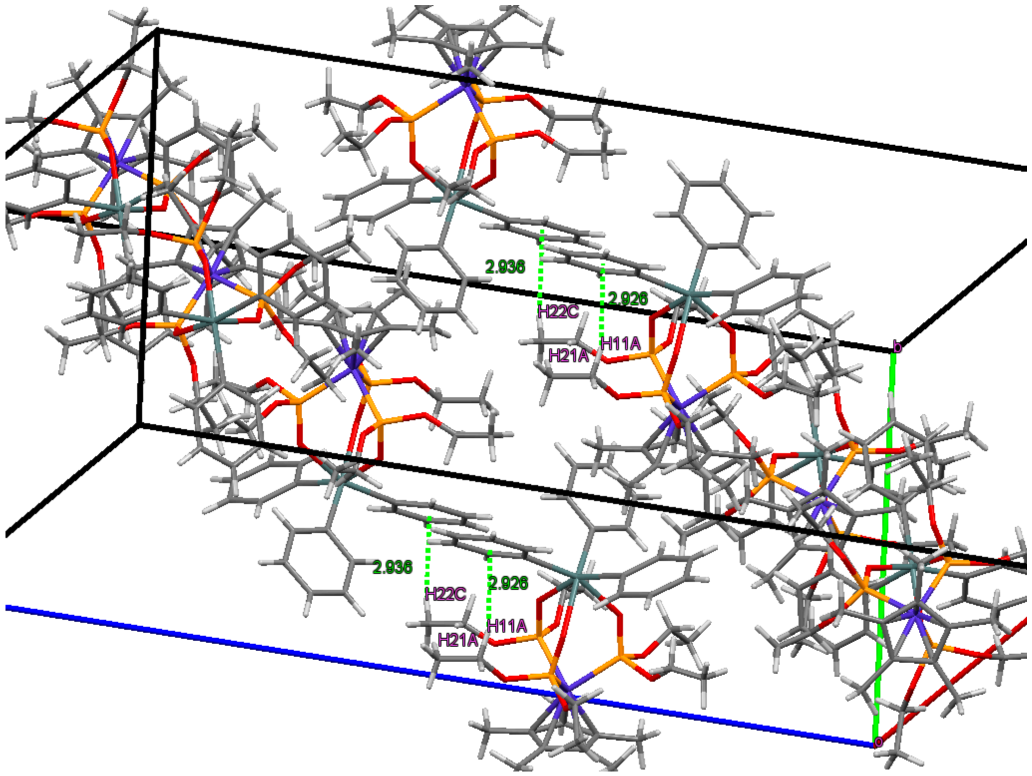

12) **Figure S11**. C–H···π interactions in **3b**

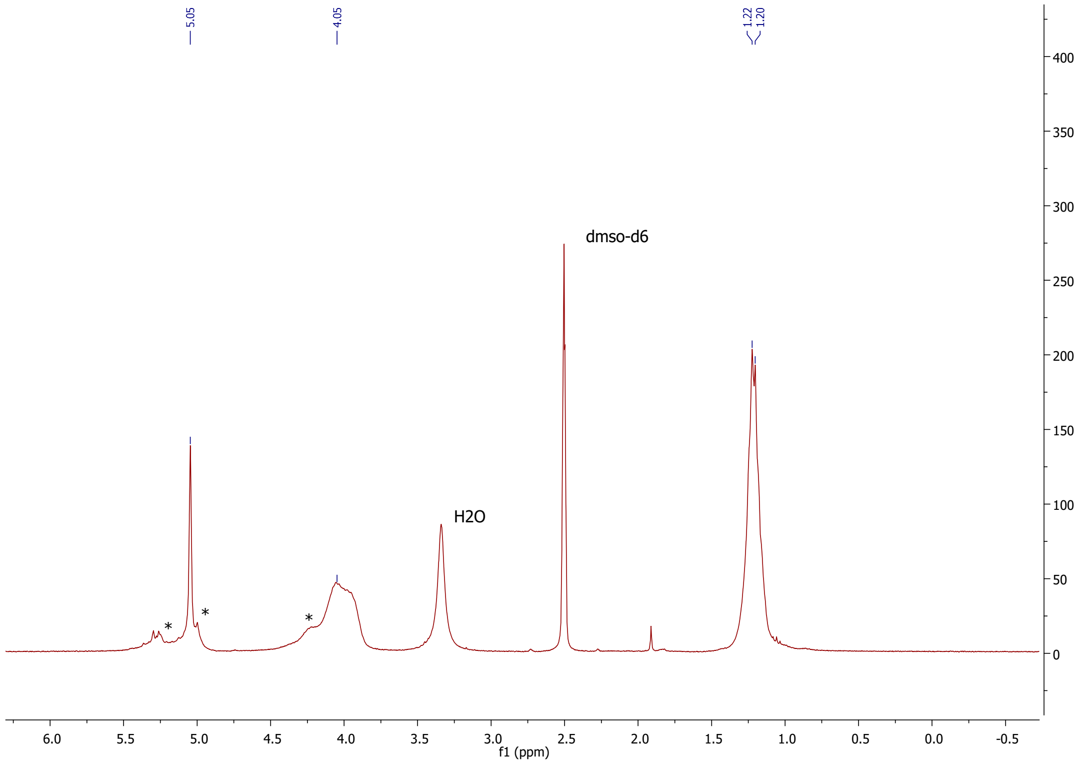

13) **Figure S12.** ^{1}H NMR of **2a** in dmso-d_6. Asterisks show the appearance of new species upon standing for hours in this solvent.

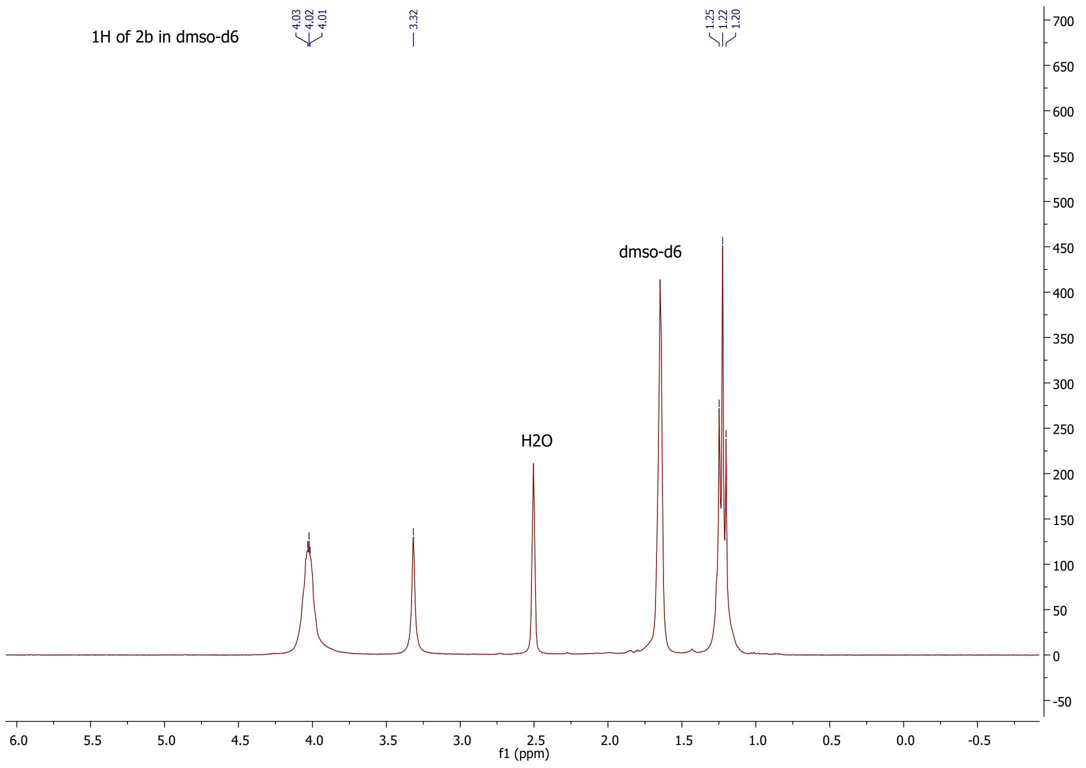

14) **Figure S13.** ^{1}H NMR of **2b** in dmso-d$_6$.

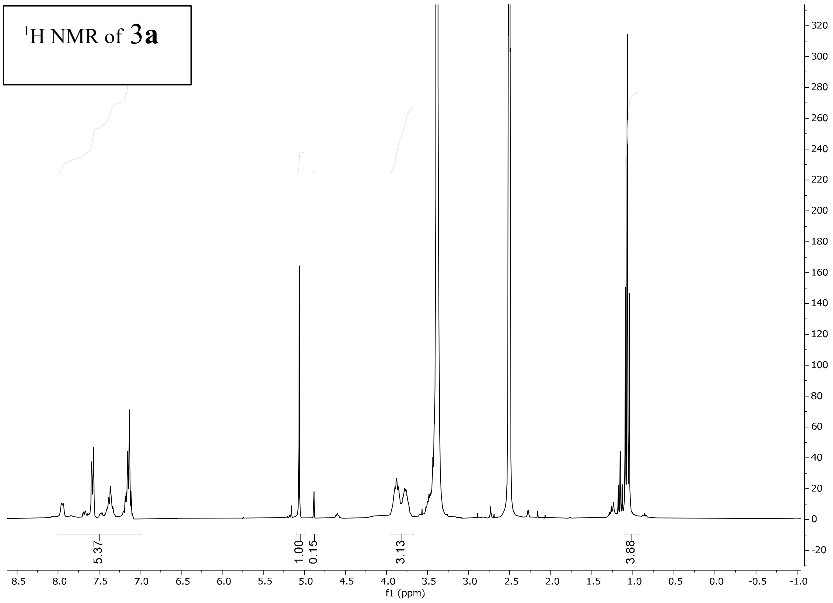

15) **Figure S14.** ^{1}H NMR spectrum of **3a** recorded in DMSO-d$_6$.

^{31}P NMR of 3**a**

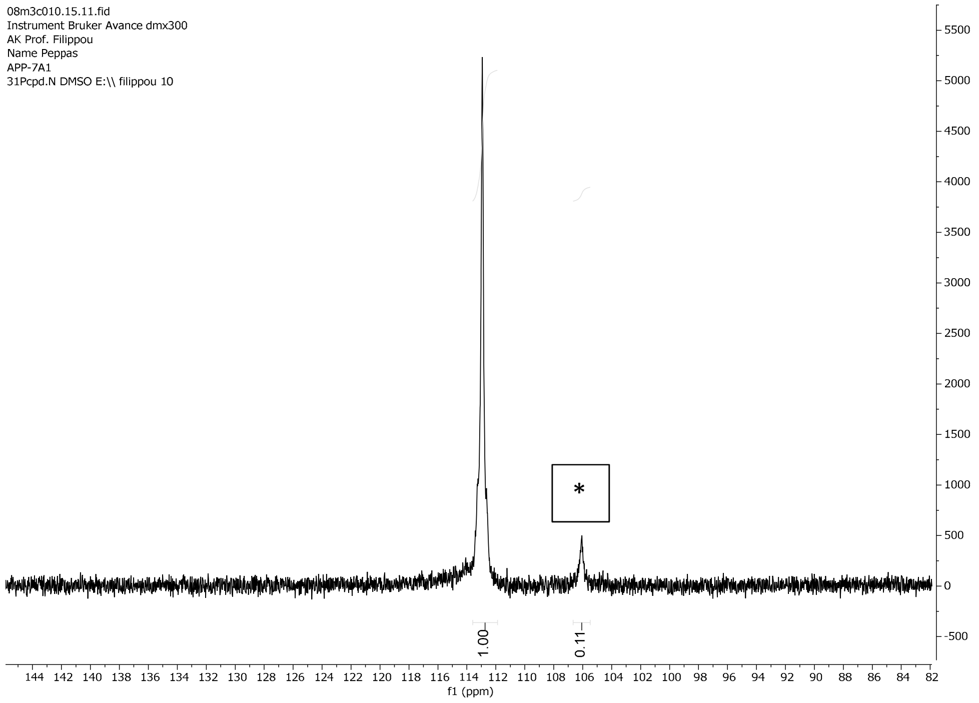

16) **Figure S15.** ^{31}P NMR spectrum of **3a** recorded in DMSO-d$_6$. Asterisk shows the appearance of new species upon standing for hours in this solvent.

17) Table S2

Table S2. Viability percentage (%) values for organotin(II) and organotin(IV) complexes against the Jurkat T lymphoblastic tumor cell line

Concentration in μM	Cisplatin	$SnCl_2{\cdot}2H_2O$	Ph_3SnCl	1a	1b	2a	2b	3a	3b
0	233.60	132.90	120.60	170.60	219.90	187.20	154.30	229.60	198.30
1	50.40	153.80	5.80	244	67.50	56.70	98.20	11.30	100
5	55.50	100.80	0	361.30	68.20	49.40	53.60	7.50	73.50
10	39.50	158.80	0	229.40	67.70	54.50	6.30	6.30	65.20
20	43.80	73.90	0	152.80	87.70	48.10	4.40	0	48.80
50	22.80	37.80	0	235	99.10	53.80	0	0	22.60
75	4.80	27.20	0	128.80	102.30	39.90	0	0	19.10

18) Molecular docking Studies

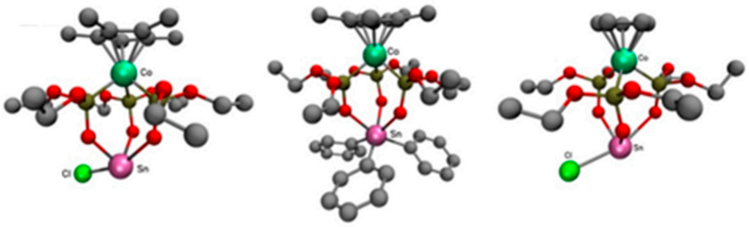

2b 3b 2a

Molecular docking calculations were performed with the aim to obtain structural insights into the interaction of the organotin complexes with PAFR. Docking of the three complexes **2a**, **2b** and **3b** were performed as described in previously [1], revealing that none of the complexes can easily fit inside the PAF-binding site of the receptor. In particular, **3b** is predicted to exhibit the highest positive free energies of binding (> 100 Kcal/mol) due to extended van der Waals clashes with PAFR. The less bulky complexes **2a** and **2b** still displayed positive free energies of binding, but in the order of 10-20 Kcal/mol, and 1-10 Kcal/mol, respectively. However, it should be noted that docking calculations were performed with a rigid model of the receptor. Therefore, a possible reorganization of the PAF-binding site residues might provide the space required to accommodate these complexes. In such case, complexes **2a** and **2b** shall be the most favorable due to the presence of the less bulky ligands. On the other hand, there is also a possibility that these complexes can interact with the extracellular domain of the receptor, thus inhibiting PAFR by blocking the entrance of PAF inside its receptor.

19) Details of the experimental protocol for biological experiments on washed rabbit platelets (WRPs)

More specifically, 250 μL of washed rabbit platelets' suspensions were added to an aggregometer cuvette, at 37 °C, stirred at 1200 rpm, and calibrated prior to testing with a blank. The maximum-reversible (or the minimum-irreversible) PAF-induced/thrombin-induced platelet aggregation was determined as the 100%

aggregation, that was also used as baseline (0% inhibition), by adding PAF at approximately 1- 5×10^{-11} M final concentration in the cuvette for WRPs and 0.1-5 x 10^{-8} M final concentration in the cuvette for rPRP or active thrombin at approximately 0.001-0.04 IU/mL for WRPs and 0.05-0.1 IU/mL for rPRP in the cuvette. The PAF-induced/thrombin-induced aggregation was calculated first at this zero % inhibition baseline in a cuvette. Then, to assess the ability of each sample to inhibit PAF/Thrombin induced platelet aggregations, WRPs or rPRP were pre-incubated -in different cuvettes- in the presence of a variety of concentrations of a tested sample, and after 1-2 min of this incubation the appropriate amount of PAF/thrombin (needed for maximum reversible platelet aggregation) was added in these cuvettes and the reduced aggregation was calculated. Consequently, a linear curve at the range of 20–80% inhibition against PAF-induced/thrombin-induced aggregation of hPRP to the concentrations of each sample was deduced. From this curve, the concentration (μM) of each sample that led to 50% of inhibition of rabbit platelet aggregation induced by PAF or thrombin in WRPs or rPRP suspensions of 0.250 mL, was calculated as the 50% inhibitory concentration value (i.e., IC_{50}) for each sample. The resulting IC_{50} values were expressed as a mean value of the concentration of each compound (μM) in the aggregometer cuvette ± standard deviation.

References

[1]. Tsoupras, A.B., Papakyriakou, A.; Demopoulos, A.C.; Philippopoulos, A.I. Synthesis, biochemical evaluation and molecular modeling studies of novel rhodium complexes with nanomolar activity against Platelet Activating Factor. *J. Inorg. Biochem.* **2013**, *120*, 63-73. doi.org/10.1016/J.JINORGBIO.2012.12.004.
